# Effectiveness of an educational intervention on knowledge about anticoagulant therapy: a quasi-experimental study

**DOI:** 10.1590/0034-7167-2025-0237

**Published:** 2026-07-27

**Authors:** Monique Oliveira do Nascimento, Érica Laryssa Lemos Souza, Isabelle Francielle Bezerra Barbosa, Betânia da Mata Ribeiro Gomes, Alexsandro Silva Coura, Thiago Moura de Araújo, Thais de Oliveira Gozzo, Simone Maria Muniz da Silva Bezerra

**Affiliations:** I Universidade de Pernambuco. Recife, Pernambuco, Brazil; II Universidade Estadual da Paraíba. Campina Grande, Paraíba, Brazil; III Universidade da Integração Internacional da Lusofonia Afro-Brazileira. Redenção, Ceará, Brazil; IV Universidade de São Paulo. Ribeirão Preto, São Paulo, Brazil

**Keywords:** Nursing, Anticoagulants, Knowledge, Health Education, Remote Patient Monitoring., Enfermería, Anticoagulante, Conocimiento, Educación en Salud, Monitorización Remota de Pacientes.

## Abstract

**Objectives:**

to assess the effectiveness of a nursing education intervention on patients’ knowledge about anticoagulant therapy.

**Methods:**

a quasi-experimental study was conducted with 25 patients using warfarin. The intervention, based on the Self-Care Deficit Nursing Theory and applied using the Supported Self-Care methodology, consisted of remote follow-up with identification of difficulties in self-care, counseling, development of a care plan, assistance, and monitoring of results. Therapeutic knowledge was assessed before and after the follow-up. The results were compared using the Wilcoxon test.

**Results:**

the comparison of knowledge scores before and after the intervention showed an increase from 5.24 to 7.74 (p<0.001). After follow-up, no participant demonstrated insufficient knowledge.

**Conclusions:**

the educational intervention proved effective in increasing knowledge about the treatment. This finding demonstrates the potential of qualified nursing care through telehealth.

## RESUMEN

**Objetivos:**

evaluar la efectividad de una intervención educativa de enfermería sobre el conocimiento de los pacientes acerca de la terapia anticoagulante.

**Métodos:**

se realizó un estudio cuasiexperimental con 25 pacientes que utilizaban Warfarina. La intervención, basada en la Teoría General del Autocuidado y aplicada mediante la metodología de Autocuidado Asistido, consistió en seguimiento remoto con identificación de dificultades en el autocuidado, asesoramiento, elaboración de un plan de cuidados, asistencia y monitorización de resultados. Se evaluó el conocimiento terapéutico antes y después del seguimiento. Los resultados se compararon mediante la prueba de Wilcoxon.

**Resultados:**

la comparación de las puntuaciones de conocimiento antes y después de la intervención mostró un aumento de 5,24 a 7,74 (p<0,001). Tras el seguimiento, ningún participante demostró conocimientos insuficientes.

**Conclusiones:**

la intervención educativa resultó eficaz para aumentar el conocimiento sobre el tratamiento. Este hallazgo demuestra el potencial de la atención de enfermería cualificada mediante la telemedicina.

## INTRODUCTION

The prescription of oral anticoagulants (OACs) has been increasing significantly in recent years, coinciding with a growing prevalence of cardiovascular conditions that commonly present with coagulopathies^(1,2)^. These drugs work by increasing clotting time, but their main side effect is the occurrence of hemorrhagic events, which requires careful management of their therapy^(3)^.

Warfarin is a vitamin K antagonist anticoagulant widely used due to its low cost, in addition to clinical factors that contraindicate the use of newer anticoagulants^(4)^. However, the narrow therapeutic range of this drug, as well as its greater predisposition to drug and food interactions, makes outpatient monitoring of patients essential for monitoring plasma coagulation time, represented by the International Normalized Ratio (INR), in addition to dosage adjustments^(1)^.

Several factors hinder a patient’s ability to reach and maintain a therapeutic INR range, such as genetics, socioeconomic and educational level, clinical conditions, and factors related to individuals’ behavior^(5,6)^. From a behavioral standpoint, noteworthy factors include the decision to adhere to medication, forgetting doses, dietary restrictions, cessation of alcohol consumption, vigilance regarding interactions with other drugs, and frequent monitoring^(7)^. Therefore, understanding the importance of warfarin treatment and its specificities is one of the essential elements for its success.

However, it is common to find inadequate levels of knowledge among patients treated with warfarin^(6)^, a factor that can lead to ineffective adherence to treatment^(8)^. In this scenario, the main knowledge gaps for these patients relate to what to do if a dose is missed, the consequences of an INR higher or lower than the target, identification of complications, and uncertainty about food and drug interactions^(7)^.

Considering the complexity of managing anticoagulant treatment, nurses’ role is undeniably relevant in promoting self-care through health education. This professional’s training includes developing skills and competencies focused on health education for patients and their families, utilizing elements such as communication, awareness, and autonomy to fulfill the role of educator^(9,10)^.

Despite the emphasis on the role of nursing in conducting educational actions to change risky behaviors, promote health, and manage therapy, in the last five years, no published studies have been found that assess the effectiveness of educational interventions conducted by nurses on clinical outcomes in the context of oral anticoagulation. However, some research indicates that educational interventions applied by healthcare professionals yield positive results in therapeutic knowledge, adherence, treatment satisfaction, and coagulation control^(11-14)^.

Some strategies used in public health to address chronic conditions may be useful in this context, such as Supported Self-Care (SSC), which aims to systematically provide educational and support interventions to increase patients’ confidence and skills in managing their health problems and their respective treatments^(15)^. This process involves regular monitoring of individuals, setting goals, and developing a personalized care plan^(16)^.

In this sense, the Educational Support System (ESS), addressed in the Nursing Systems Theory, which comprises Dorothea Orem’s Self-Care Deficit Nursing Theory (SCDNT), corroborates the proposal to encourage and guide patients’ self-care. In this theory, the ESS considers individuals’ preserved self-care capacity, directing nursing care towards supporting and guiding the patient in performing specific care tasks^(17)^.

Therefore, considering the deficiencies in knowledge about OAC therapy and its unfavorable clinical consequences, the use of educational interventions becomes pertinent, as well as assessing their effect on clinical outcomes of anticoagulation. This study aims to assess the effectiveness of a nursing educational intervention in patients’ knowledge about anticoagulant therapy.

## OBJECTIVES

To assess the effectiveness of a nursing education intervention on patients’ knowledge about anticoagulant therapy. The hypothesis considered in this study is that SSC via teleconsultation increases knowledge about anticoagulant therapy in patients chronically using warfarin.

## METHODS

### Ethical aspects

The research was conducted following the ethical guidelines for research involving human subjects, having been approved by the *Universidade de Pernambuco* Research Ethics Committee and by the Brazilian Registry of Clinical Trials - RBR-3ms5dgd.

Potential adverse events associated with the intervention included discomfort or embarrassment in answering certain questions about personal aspects and the treatment. In order to minimize these discomforts, the researchers prepared to adopt measures such as interrupting the interview and providing immediate and subsequent emotional support. Despite this, none of the patients interviewed and followed up in the research presented any adverse event.

### Study design, period and location

This is a quasi-experimental, single-group before-and-after study without allocation, where the unit of analysis was the participant in paired measures. There was no blinding during participation, intervention application, or analysis. The description of this study was based on the Enhancing the Quality and Transparency of Health Research Network Transparent Reporting of Evaluations with Nonrandomized Designs protocol, used in public health interventions with non-randomized designs^(18)^. The investigation was conducted with patients from the oral anticoagulation outpatient clinic of a reference cardiology hospital in the state of Pernambuco from September 2022 to August 2023.

### Sample, inclusion and exclusion criteria

The sample consisted of 25 individuals undergoing warfarin treatment. Sample size calculation was performed using the paired single-sample t-test. An effect size of 20% after the intervention was considered, with a standard deviation of 1.8 obtained from a previous study^(19)^, a standardized effect size of 0.90, a two-tailed test with 80% power and a significance level of 5%, totaling 11 patients. Adding at least 25% to account for losses to follow-up, a minimum of 15 participants would be required.

Inclusion criteria comprised being at least 18 years old, undergoing warfarin treatment, being followed up at the anticoagulation outpatient clinic, obtaining an unsatisfactory score (≤8) on the knowledge scale about anticoagulant treatment, presenting a satisfactory cognitive assessment according to the Mini-Cog^©^ test application^(20)^, and having a landline or mobile phone available for remote follow-up. Exclusion criteria comprised pregnant women, any communication barriers that would limit the intervention, memory impairment, and a desire to withdraw from follow-up.

**Figure 1:**
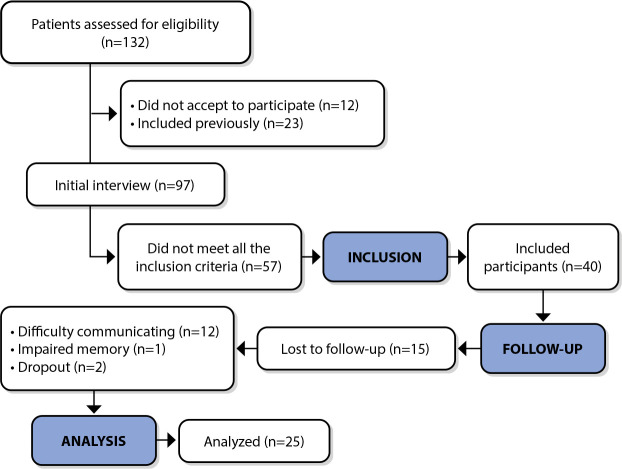
Flowchart of participants in the study, Recife, Pernambuco, Brazil, 2023

The sampling process was non-probabilistic, based on convenience, considering that there were few patients at the study site who met all the inclusion criteria. A total of 132 patients were approached, of whom 97 proceeded to an initial interview. Of these, 40 were included in the study after meeting all the inclusion criteria. During follow-up, 15 patients were excluded. Figure 1 presents the participant flowchart.

### Study protocol

#### Recruitment and presentation of Informed Consent Form and other instruments

To recruit participants, active outreach was conducted weekly at the hospital’s anticoagulation clinic. After explaining the study, participants were invited to participate, accepting the offer by signing the Informed Consent Form, followed by an assessment of their level of knowledge about anticoagulant treatment, as well as a cognitive assessment. A sociodemographic and clinical questionnaire was administered to patients who met all inclusion criteria.

**Figure 2:**
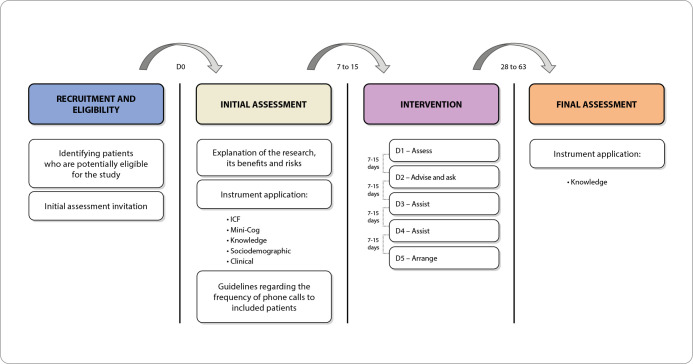
Main stages of study development, Recife, Pernambuco, Brazil, 2023

During recruitment, participants were instructed on how to receive phone calls as part of the study intervention. They were asked about their preferred day and time for this phone contact, as well as their availability to use WhatsApp to receive messages or calls (Figure 2).

#### Educational intervention and outcome reassessment

The intervention took place via telephone contact and was conducted by a nurse trained to apply an SSC promotion script for patients chronically using OACs, which had undergone prior content validity with specialists. A smartphone with a SIM card exclusively for research use was employed. Contact attempts were always made in a quiet environment, on the days and times indicated by participants, and when contact was unsuccessful, the attempt was repeated once more on the same day.

During the educational intervention, efforts were made to respect each individual’s beliefs and prior knowledge, as well as the self-care deficits identified during the assessment phase, in order to meet their real needs. From this perspective, the application of SSC protocol was guided by the ESS, and included activities such as teaching, guiding, supervising, and providing a supportive environment for the development of self-care in anticoagulant treatment, which are strategic axes in Systems Theory^(17)^.

Furthermore, the educational intervention actions were structured for systematic application based on the 5As method. The first day of intervention (D1) corresponded to the self-care assessment stage, with an estimated duration of about seven minutes; on the second day (D2), the counseling and agreement stages were applied, with a time of approximately 20 minutes; the third and fourth days of intervention (D3 and D4) were planned to offer assistance with difficulties in complying with the agreement made on the second day, with an approximate duration of 15 minutes each day; finally, on the last day of follow-up (D5), the self-care plan was monitored with a new knowledge assessment, with a telephone call time of approximately eight minutes. In total, five completed calls were made to each participant, with an estimated duration of 65 minutes, distributed among the completed phone calls.

Chart 1 presents the actions taken to promote self-care among patients using OACs.

**Chart 1:** Supported Self-Care Actions for anticoagulated patients according to the 5As method and intervention days, Recife, Pernambuco, Brazil, 2023

DAYS OF INTERVENTION	5AS METHOD STAGE	ACTIONS	
**D1**	**Assess**	1	Ask what is most important to individuals in self-care with anticoagulant treatment.
		2	Ask what barriers individuals perceive to their self-care in the context of their treatment with OACs.
		3	Assess the importance and degree of confidence individuals have in developing self-care.
**D2**	**Advise**	1	Inform/reinforce information about individuals’ clinical condition indicating the need for anticoagulation, about warfarin treatment, and about the meaning of their INR results.
		2	Inform individuals about the importance of following the medication and behavioral treatment of oral anticoagulation.
		3	Inform about which behavioral changes and care are recommended in the context of oral anticoagulation.
		4	Encourage individuals to make behavioral changes and to perform the necessary care in anticoagulant treatment.
		5	Suggest ways to operationalize behavioral changes, self-care, and management of OACs.
	**Agree**	1	Agree with individuals on specific self-care goals for anticoagulant treatment.
		2	Encourage individuals to seek help from relatives and friends to achieve the goals.
		3	List and discuss the benefits of the agreed-upon goals.
		4	Establish, collaboratively, an action plan to achieve the defined goals.
		5	Establish, collaboratively, the person’s level of confidence in achieving the goal.
**D3 and D4**	**Assist**	1	Assist the person identify the barriers to achieving their established self-care goals.
		2	Identifying, with individuals, the resources available in the family, the community, and in terms of available technologies that can support self-care.
		3	Discuss the self-care plan with individuals, reviewing progress and reassessing goals.
		4	Use motivational strategies to ensure the continuity of the self-care plan.
**D5**	**Arrange**	1	Monitor, together with individuals, compliance with the self-care plan.
		2	Assess the results of the level of knowledge about anticoagulant treatment.
		3	Check the need for adjustments to the care plan.

*OACs - oral anticoagulants; INR - International Normalized Ratio; D - day.*

*Source: Whitlock et al. (2002). Adapted.*

The interval adopted between each call/intervention day was seven to 15 days, considering that some contact attempts were unsuccessful. Thus, the mean follow-up time for participants was 38 days (±8.5), with the shortest and longest follow-up times being 28 and 63 days, respectively.

In addition to phone calls to study participants, they also received WhatsApp or SMS messages, and were asked about their preference for written or audio messages. The content of these messages was developed by two specialist nurses with experience in the anticoagulation clinic. The messages were further adapted for better patient comprehension, after assessment and suggestions from an undergraduate nursing student.

The messages were personalized according to the self-care difficulties identified in each individual on the first day of intervention (D1). This communication via message allowed for the reinforcement of information received by participants during counseling, focusing on the areas most difficult to understand. The messages also reinforced the goals agreed upon in the self-care plan, as well as serving as a means of motivational support for self-care continuity.

During the follow-up period, each participant received two weekly scheduled messages, with a minimum interval of three days. Individuals frequently responded to the messages sent to them or sent messages to clarify doubts or report difficulties. In these situations, they received a response within 24 hours.

Knowledge assessment was conducted using an instrument that measures this outcome in patients using OACs, which was duly translated and validated for Portuguese^(21)^. The questionnaire consists of 11 questions, with the answer options being “does not know”, “knows partially”, or “knows”, with values of zero, half a point, and one point assigned to each answer, respectively. Based on the quantification and summation of the answers, the following classification was obtained: insufficient knowledge (up to four points); regular knowledge (from five to eight points); and adequate knowledge (above eight points).

#### Analysis of results and statistics

For statistical processing, the Statistical Package for the Social Sciences version 26.0 (IBM Corp., Armonk, NY, USA), RStudio 2025.09.0, and STATA 12.0 were used. Descriptive statistical tools were employed. Differences in baseline characteristics between retained and excluded individuals were assessed using the chi-square test for categorical variables and the Mann-Whitney test for continuous variables.

For comparison of paired means, the Bootstrap method was used to increase the robustness of the analysis and minimize possible sampling biases. The 95% Confidence Intervals for the difference between the means were estimated considering the resampling procedure using Bootstrap BCa (Bias-Corrected and Accelerated), which provides greater statistical precision, especially in small sample sizes. The magnitude of the intervention effect was expressed by Cohen’s d for paired data, a standardized indicator of the practical relevance of the observed differences. The 95% Confidence Interval for Cohen’s d was also calculated based on Bootstrap BCa, ensuring greater reliability of the estimates.

To assess the marginal homogeneity between knowledge categories before and after the intervention, the Stuart-Maxwell test was used. This test allows us to verify whether the marginal distributions of categories remain equivalent over time. All tests were applied with a significance level of 5% (p-value ≤ 0.05), and no additional analyses were performed.

## RESULTS

Twenty-five patients on chronic warfarin therapy were followed. When comparing sociodemographic characteristics between retained (n=25) and excluded (n=15) patients, no statistically significant differences were observed regarding mean age (58 years ± 11.7 versus 63.60 ± 13.27 years, p=0.154), female sex (60% versus 46.7%; p=0.412), non-white race (80% versus 93.3%; p=0.381), marital status in a conjugal union (68% versus 66.7%; p=1.000), absence of employment (92% versus 73.3%; p=0.174), mean education (6.72 ± 3.47 versus 7.73 ± 5.87 years of study; p=0.622) and mean monthly *per capita* family income (561.44 ± 351.50 versus 717.02 ± 612.72 reais; p=0.705).

Concerning baseline clinical variables, homogeneity was also observed between both groups, such as medication costs (92% versus 93.3%; p=1.000), atrial fibrillation as the most frequent clinical indication for anticoagulation (56% versus 60%; p=0.804), and median treatment time (4 [p25=2; p75=9] versus 3 [p25-2; p75-5] years; p=0.287), episodes of hemorrhagic (28% versus 33.3%; p=0.736) or thromboembolic (20% versus 26.7%; p=0.705) complications, self-reported treatment interruption (24% versus 20%; p=1.000), receiving professional guidance on anticoagulant therapy (56% versus 53.3%; p=0.870), doubts about treatment (36% versus 53.3%; p=0.283), and mean knowledge score about treatment (5.24 ± 1.38 versus 4.63 ± 2.01%; p=0.249).

Participants’ knowledge was assessed at two points in time when this outcome was measured (before and after the intervention). Table 1 shows the total knowledge, partial knowledge, and lack of knowledge about points relevant to anticoagulant treatment. The most frequent knowledge deficit before the intervention was regarding the medication’s side effects, the necessary care during treatment, the target INR value, factors that interfere with INR stability, the consequences of not using the medication, as well as the clinical indication for treatment. Follow-up led to lower percentages of knowledge deficit.

**Table 1:** Patients’ knowledge about oral anticoagulant therapy before and after the intervention (N = 25), Recife, Pernambuco, Brazil, 2023

QUESTIONS	Knows n (%)		Knows partially n (%)		Does not know n (%)	
	Before	After	Before	After	Before	After
1. What is the name of the anticoagulant you are taking?	21 (84)	23 (92)	01 (04)	-	03 (12)	02 (08)
2. Do you know what this medication is for?	15 (60)	21 (84)	08 (32)	03 (12)	02 (08)	01 (04)
3. Do you know why you are taking this medication?	09 (36)	15 (60)	06 (24)	05 (20)	10 (40)	05 (20)
4. Do you know what the side effects of anticoagulants are? (at least 1)	01 (04)	16 (64)	01 (04)	-	23 (92)	09 (36)
5. What dose of OACs are you currently taking? Please tell.	20 (80)	23 (92)	02 (08)	01 (04)	03 (12)	01 (04)
6. How long have you been taking OACs?	16 (64)	16 (64)	08 (32)	07 (28)	01 (04)	02 (08)
7. What can happen if you do not take the OACs?	07 (28)	15 (60)	08 (32)	01 (04)	10 (40)	09 (36)
8. What is your target INR?	10 (40)	15 (60)	01 (04)	07 (28)	14 (56)	03 (12)
9. Do you know what factors can interfere with INR levels? (at least 1)	11 (44)	18 (72)	01 (04)	01 (04)	13 (52)	06 (24)
10. Do you know what precautions you need to take when using OACs? (at least 2)	01 (04)	12 (48)	10 (40)	11 (44)	14 (56)	02 (08)

*OAC - oral anticoagulant; INR - International Normalized Ratio.*

As shown in Table 2, the mean scores of participants’ knowledge about treatment increased by more than two points after the nursing intervention. This difference showed statistical significance (p<0.001) with a significant effect size (d = 1.53; 95%CI: 1.07 - 2.04). When comparing the outcome categories before and after the intervention, a positive shift between knowledge categories is observed (from insufficient to regular and satisfactory). The Stuart-Maxwell marginal homogeneity test showed a statistically significant difference between the marginal distributions of the two time points (p<0.001), indicating that participants’ knowledge profile underwent a relevant change over time.

**Table 2:** Knowledge score regarding anticoagulant therapy before and after the intervention (N = 25), Recife, Pernambuco, Brazil, 2023

KNOWLEDGE								
	**Before**		**After**					
	**n (%)**	**Mean score** **(±SD)**	**n (%)**	**Mean score** **(±SD)**	95%CI of the difference^**^	*p* value^*^	**Cohen’s d (paired)**	**95%CI** Cohen ^**^
Insufficient	09 (36)	5.24 (±1.38)	-	7.74 (±1.24)	1.90 - 3.14	**<0.001**	1.53	1.07 - 2.04
Regular	16 (64)		14 (56)					
Satisfactory	-		11 (44)					

* Paired mean comparison using the Bootstrap method;

** 95%CI (Bootstrap BCa); 95%CI - 95% Confidence Interval; SD - standard deviation.

Some of the self-care actions suggested to patients are: organizing their daily routine, as well as using alarms and family help to avoid forgetting to take medication; increasing knowledge about food, drug, and herbal medicine interactions; regular INR monitoring with in-person consultations or teleconsultations; meal planning; using protective equipment in activities with a risk of cuts or bleeding; and dialogue with the healthcare team to seek possible alternatives for health treatment in the face of financial difficulties in acquiring medications.

## DISCUSSION

In the results regarding knowledge of aspects related to treatment with OACs, it was observed that, after the intervention, adequate knowledge was achieved in most areas, with particular emphasis on increased knowledge about the side effects of the medication and the necessary precautions when using OACs.

When comparing the mean outcome score before and after the intervention, a positive impact on therapeutic knowledge is observed. A quasi-experimental study, conducted in an anticoagulation outpatient clinic, found that, after follow-up with periodic visits and counseling on anticoagulant medication, there was a significant increase in the knowledge score about treatment^(19)^.

Remote educational interventions using technology have also proven to be viable options with a significant impact on patients’ knowledge of their therapy. A systematic review showed improved knowledge levels among patients who received educational and self-management instructions in the context of anticoagulation through mobile applications on smartphones or tablets^(11)^.

Additionally, it is worth highlighting the prominence that telehealth has gained, especially after the COVID-19 pandemic, demonstrating its ability in many situations to guarantee the continuity of healthcare for patients remotely. This resource is capable of generating positive impacts on clinical outcomes, as well as more flexible access to the healthcare system. In this context, there are projections for the continued use of telehealth and its likely expansion as a complementary format to in-person patient care^(22)^.

Therefore, reports of the use of technologies applied to anticoagulant treatment have become increasingly common, with satisfactory results both in healthcare continuity and in improving levels of knowledge, therapeutic adherence, satisfaction, and clinical outcomes. These technological approaches are implemented through health education programs via teleconsultation, text messaging, and mobile applications^(12,13)^.

Strategies for repeating advice within the context of health education, including through text messages, have been cited in the literature as ways to better consolidate patients’ knowledge^(6,12)^. In this way, it can be inferred that both the telephone reinforcement of instructions on warfarin treatment and the periodic weekly sending of text messages to patients in this study were likely contributing factors to achieving more satisfactory levels of knowledge.

From this perspective, an educational experience and active patient participation, including discussion of negative experiences with medication and treatment, can strengthen knowledge and adherence to anticoagulant therapy^(8)^.

Regarding the literature’s approach to SSC in anticoagulation, international studies show that the way this topic is presented generally focuses on self-care for the purpose of self-testing and self-management of medication doses^(14,23)^. This differs from the Brazilian approach, whose focus on promoting self-care is more directed towards general care without involving more complex aspects of treatment^(24)^.

Regarding the systematic use of SSC for anticoagulated patients, two studies with this approach were found. A study conducted in Turkey used systematized SSC within the 5As method as part of a home care program developed by nurses for patients using warfarin, obtaining positive effects on self-management and patient satisfaction^(14)^.

Another study was developed in Brazil, corresponding to the construction and validity of a protocol to promote behavioral change in patients using warfarin, seeking their active participation in self-care. Although it did not cite the 5As method as its basis, the protocol created includes some of the stages of that method, such as patient assessment with problem definition, goal setting, development of a care plan, and assistance in achieving the goals agreed upon in the plan^(24)^.

From the perspective SCDNT, Orem mentions the specific self-care requirements in the face of health deviations. This specificity was designed for people who have some illness, disability, or disorder with particular care needs^(17)^. From this perspective, it is understood that people with chronic health conditions commonly require a specific self-care routine, considering the demands that arise during the course of their illness^(25)^. Patients on anticoagulants also require specific care, given the complexity of their treatment.

Furthermore, it is important to highlight the importance of the bond between the healthcare team, individuals, and their family as a guiding thread within the process of promoting self-care and providing educational support from nurses to achieve favorable outcomes. Therefore, building a bond implies responsibility between professionals and individuals, resulting from the coexistence of elements such as effective communication, establishing trust, and fostering affective relationships^(25)^.

### Study limitations

This study had some limitations regarding sample size, as the researchers encountered saturation of eligible individuals within the studied population, in addition to the infeasibility of randomizing the sample.

Furthermore, it is pertinent to highlight the biases that can affect the study’s internal validity. Some of these biases are: regression to the mean, minimized by the presence of varying initial knowledge scores among patients; test effect, attenuated by the minimum time interval of four weeks between assessments and by the conceptual nature of the questionnaire; fidelity in the implementation of the intervention, which was ensured by following a protocol for conducting the educational intervention and by external monitoring; and history and maturation bias, which may have influenced the positive results of the intervention, although the short follow-up time suggests a low impact on participants’ natural maturation.

With regard to external validity, the generalizability of results must consider the intervention frequency and standardization according to protocol, the language of the educational intervention adapted to the education and local culture, access to technological resources for communication, and the conduct of the study by a team linked to a reference hospital in cardiology, whose support is different from that offered in less complex services.

In light of the above, it is recommended that further studies with randomized designs be conducted in more than one healthcare facility to test the application of SSC in anticoagulated patients and its clinical benefits.

### Contributions to health and nursing

The findings of this research, regarding the positive impact of SSC via teleconsultation conducted by a professional nurse, may serve as a basis for the application and improvement of this care model in the outpatient follow-up of individuals using OACs, as well as in the context of other chronic conditions.

Furthermore, they expand the possibilities for nursing practice through communication technologies, enabling the provision of qualified care while optimizing available resources. Maintaining and strengthening the bond with patients through telehealth has therefore highlighted the potential of this tool in assisting individuals undergoing anticoagulant treatment.

## CONCLUSIONS

The nursing education intervention had a positive impact on patients’ knowledge about anticoagulant therapy, indicating its effectiveness. Therefore, the study points to the potential for success in conducting health education programs, led by nurses and carried out remotely with anticoagulated patients.

## Data Availability

The research data are available within the article.
