# Supplementary material for: Effectiveness of an educational intervention on knowledge about anticoagulant therapy: a quasi-experimental study
Source: Rev Bras Enferm. 2026 Jul 27;79:e20250237. doi: 10.1590/0034-7167-2025-0237 (PMC13411317; doi:10.1590/0034-7167-2025-0237)
Supplement: Translation

# Translation: Efetividade de uma intervenção educativa no conhecimento sobre a terapia anticoagulante: estudo quase-experimental

**DOI:** 10.1590/0034-7167-2025-0237pt

**Authors:** Monique Oliveira do Nascimento, Érica Laryssa Lemos Souza, Isabelle Francielle Bezerra Barbosa, Betânia da Mata Ribeiro Gomes, Alexsandro Silva Coura, Thiago Moura de Araújo, Thais de Oliveira Gozzo, Simone Maria Muniz da Silva Bezerra

**Affiliations:** I Universidade de Pernambuco. Recife, Pernambuco, Brasil; II Universidade Estadual da Paraíba. Campina Grande, Paraíba, Brasil; III Universidade da Integração Internacional da Lusofonia Afro-Brasileira. Redenção, Ceará, Brasil; IV Universidade de São Paulo. Ribeirão Preto, São Paulo, Brasil

**Keywords:** Enfermagem, Anticoagulante, Conhecimento, Educação em Saúde, Monitoramento Remoto de Pacientes.

## Abstract

**Objetivos:**

avaliar a efetividade de uma intervenção educativa de enfermagem no conhecimento dos pacientes sobre a terapia anticoagulante.

**Métodos:**

estudo quase-experimental, realizado com 25 pacientes em uso de varfarina. A intervenção, fundamentada na Teoria Geral do Autocuidado e aplicada pela metodologia do Autocuidado Apoiado, consistiu em um acompanhamento remoto com identificação das dificuldades para o autocuidado, aconselhamento, elaboração de um plano de cuidados, assistência e monitoramento dos resultados. O conhecimento terapêutico foi avaliado antes e após o acompanhamento. Os resultados foram comparados pelo teste de Wilcoxon.

**Resultados:**

a comparação dos escores de conhecimento antes e após a intervenção apontou para um aumento de 5,24 para 7,74 (p<0,001). Após o acompanhamento, nenhum participante demonstrou conhecimento insuficiente.

**Conclusões:**

a intervenção educativa demonstrou efetividade com o aumento do conhecimento sobre o tratamento. Esse achado demonstra a potencialidade de uma assistência de enfermagem qualificada através da telessaúde.

## INTRODUÇÃO

A prescrição de anticoagulantes orais (ACOs) vem aumentando significativamente nos últimos anos, ao passo que cresce a prevalência de condições cardiovasculares que comumente cursam com coagulopatias^(1,2)^. Esses fármacos agem no aumento do tempo da coagulação, mas apresentam como principal efeito colateral a ocorrência de eventos hemorrágicos, o que demanda cuidado para o gerenciamento de sua terapêutica^(3)^.

A varfarina é um anticoagulante antagonista da vitamina K amplamente utilizado devido ao seu baixo custo, além dos fatores clínicos que contraindicam o uso dos novos ACOs^(4)^. Contudo, a estreita faixa terapêutica desse fármaco, bem como a sua maior predisposição a interações medicamentosas e alimentares, torna indispensável o acompanhamento ambulatorial dos pacientes para monitorização do tempo de coagulação plasmática, representado pelo *International Normalized Ratio* (INR), além de ajustes das dosagens^(1)^.

Alguns fatores dificultam o alcance e a permanência do paciente na faixa terapêutica do INR, a exemplo da genética, nível socioeconômico e educacional, condições clínicas, e fatores relacionados ao comportamento do indivíduo^(5,6)^. Do ponto de vista comportamental, podem-se destacar a decisão de aderir ao medicamento, o esquecimento das doses, restrições alimentares, a cessação do consumo alcoólico, a vigilância quanto às interações com outras drogas e o monitoramento frequente^(7)^. Dessa maneira, conhecer sobre a importância do tratamento com varfarina e suas especificidades representa um dos elementos indispensáveis para o seu sucesso.

Contudo, é comum encontrar níveis de conhecimento inadequados entre os pacientes tratados com varfarina^(6)^, fator que pode conduzir à adesão ineficaz do tratamento^(8)^. Nesse cenário, os principais déficits no conhecimento desses pacientes dizem respeito ao que fazer diante da dose esquecida, às consequências do INR maior ou menor do que o alvo proposto, à identificação das complicações, e à incerteza sobre as interações alimentares e medicamentosas^(7)^.

Ao considerar a complexidade do gerenciamento do tratamento anticoagulante, a atuação do enfermeiro tem relevância indiscutível na promoção do autocuidado por meio da educação em saúde. Esse profissional possui em sua formação a construção de habilidades e competências voltadas à educação em saúde dos pacientes e familiares, utilizando elementos como a comunicação, a consciência e a autonomia para desempenhar o papel de educador^(9,10)^.

Apesar do destaque ao papel da enfermagem na condução de ações educativas para mudança de comportamentos de risco, promoção da saúde e gerenciamento terapêutico, nos últimos cinco anos, não foram encontrados estudos publicados que avaliem a efetividade de intervenções educativas conduzidas por enfermeiros sobre desfechos clínicos do contexto da anticoagulação oral. Contudo, algumas pesquisas apontam que intervenções educativas aplicadas por profissionais de saúde trazem resultados positivos no conhecimento terapêutico, adesão, satisfação com o tratamento e controle da coagulação^(11-14)^.

Algumas estratégias utilizadas na saúde pública para o enfrentamento das condições crônicas podem ser úteis nesse contexto, como o Autocuidado Apoiado (AA), que visa à prestação sistemática de intervenções educacionais e de apoio para aumentar a confiança e as habilidades dos pacientes em gerenciar seus problemas de saúde e respectivos tratamentos^(15)^. Esse processo envolve o acompanhamento periódico do indivíduo, o estabelecimento de metas e a elaboração de um plano de cuidados personalizado^(16)^.

Nesse sentido, o Sistema Educativo de Apoio (SEA), abordado na Teoria dos Sistemas de Enfermagem, a qual compõe a Teoria Geral do Autocuidado (TGA) de Dorothea Orem, corrobora a proposta de incentivo e orientação ao autocuidado dos pacientes. Na referida teoria, o SEA considera a capacidade de autocuidado preservada do indivíduo, direcionando a assistência de enfermagem para apoio e orientação do paciente na execução de cuidados específicos^(17)^.

Dessa maneira, ao considerar as deficiências no conhecimento sobre a terapia com ACO e os seus desdobramentos clínicos desfavoráveis, torna-se pertinente o uso de intervenções educativas, bem como avaliar o seu efeito em desfechos clínicos da anticoagulação. O presente estudo se propõe a avaliar a efetividade de uma intervenção educativa de enfermagem no conhecimento dos pacientes sobre a terapia anticoagulante.

## OBJETIVOS

Avaliar a efetividade de uma intervenção educativa de enfermagem no conhecimento dos pacientes sobre a terapia anticoagulante. A hipótese considerada neste estudo é que o AA por teleconsulta aumenta o conhecimento sobre a terapia anticoagulante dos pacientes em uso crônico de varfarina.

## MÉTODOS

### Aspectos éticos

A pesquisa foi realizada seguindo os preceitos éticos de pesquisas com seres humanos, tendo sido aprovada pelo Comitê de Ética em Pesquisa da Universidade de Pernambuco e pelos Registros Brasileiros de Ensaios Clínicos - RBR-3ms5dgd.

Os potenciais eventos adversos atrelados à intervenção realizada abrangeram o desconforto ou constrangimento em responder a alguns questionamentos sobre aspectos pessoais e do tratamento. A fim de minimizar esses desconfortos, os pesquisadores se prepararam para adotar medidas como interrupção da entrevista e suporte emocional imediato e posterior. Apesar disso, nenhum paciente entrevistado e acompanhado na pesquisa apresentou qualquer evento adverso.

### Desenho, período e local do estudo

Trata-se de estudo quase-experimental, com grupo único antes-e-depois, sem alocação, cuja unidade de análise foi o participante em medidas pareadas. Não houve cegamento na participação, aplicação da intervenção e análise. A descrição do presente estudo foi baseada no protocolo *Transparent Reporting of Evaluations with Nonrandomized Designs* da rede *Enhancing the Quality and Transparency of Health Research Network*, utilizado nas intervenções de saúde pública em delineamentos não randomizados^(18)^. A investigação foi realizada com os pacientes do ambulatório de anticoagulação oral de um hospital cardiológico de referência no estado de Pernambuco de setembro de 2022 a agosto de 2023.

### Amostra, critérios de inclusão e exclusão

A amostra foi composta por 25 indivíduos em tratamento com varfarina. O cálculo amostral foi realizado pelo teste T para única amostra pareada. Consideraram-se uma magnitude de efeito de 20% após a intervenção, desvio padrão de 1,8 obtido por estudo prévio^(19)^, magnitude padronizada de efeito de 0,90, teste bilateral com poder de 80% e significância de 5%, totalizando 11 pacientes. Acrescentando pelo menos 25% para suprir as perdas de seguimento, seriam necessários, no mínimo, 15 participantes.

Foram estabelecidos como critérios de inclusão ter idade mínima de 18 anos, fazer tratamento com varfarina, estar em acompanhamento no ambulatório de anticoagulação, obter na escala de conhecimento sobre o tratamento anticoagulante um escore insatisfatório (≤8), apresentar avaliação cognitiva satisfatória conforme aplicação do teste Mini-Cog^©(20)^ e ter disponibilidade de telefone fixo ou celular para acompanhamento remoto. Os critérios de exclusão foram mulheres grávidas, qualquer barreira de comunicação que limitasse a realização da intervenção, comprometimento na memória e desejo de desistência do acompanhamento.

O processo de amostragem foi do tipo não probabilístico por conveniência, considerando que, no local do estudo, havia poucos pacientes que satisfizessem todos os critérios de inclusão. No total, foram abordados 132 pacientes, dentre os quais 97 seguiram para entrevista inicial. Desses, 40 foram incluídos no estudo após preencher todos os critérios de inclusão. Durante o acompanhamento, 15 pacientes foram excluídos. A Figura 1 apresenta o diagrama de fluxo dos participantes.

**Figura 1:** Diagrama de fluxo dos participantes no estudo, Recife, Pernambuco, Brasil, 2023

### Protocolo de estudo

#### Recrutamento e apresentação do Termo de Consentimento Livre e Esclarecido e instrumentos

Para recrutamento dos participantes, realizou-se busca ativa semanalmente no ambulatório de anticoagulação do hospital. Após explanação do estudo, foram convidados a participar com aceitação mediante assinatura do Termo de Consentimento Livre e Esclarecido, seguida da aferição do nível de conhecimento sobre o tratamento anticoagulante, bem como da avaliação cognitiva. Aos pacientes que satisfizeram todos os critérios de inclusão, aplicava-se o questionário sociodemográfico e clínico.

Durante o recrutamento, os participantes foram orientados sobre o recebimento de ligações telefônicas como etapa da intervenção do estudo. Os mesmos foram consultados quanto ao dia e horário de preferência para esse contato telefônico, bem como quanto à disponibilidade de uso de *WhatsApp* para recebimento das mensagens ou ligações (Figura 2).

**Figura 2:** Etapas principais de desenvolvimento do estudo, Recife, Pernambuci, Brasil, 2023

#### Intervenção educativa e reavaliação do desfecho

A intervenção ocorreu por contato telefônico, e foi conduzida por uma enfermeira treinada para aplicar um roteiro de promoção do AA para pacientes em uso crônico de ACO, o qual passou pelo processo de validação de conteúdo prévia com especialistas. Utilizou-se um *smartphone* com *chip* de uso exclusivo da pesquisa. As tentativas de contato ocorreram sempre em ambiente silencioso, nos dias e horários sinalizados pelos participantes, e quando não havia êxito no contato, a tentativa era repetida mais uma vez no mesmo dia.

Durante a realização da intervenção educativa, buscou-se respeitar as crenças e os saberes prévios de cada indivíduo, bem como os déficits de autocuidado encontrados na etapa da avaliação, de forma a atender às suas reais necessidades. Nessa perspectiva, a aplicação do roteiro de AA foi balizada pelo SEA, e contemplou as atividades de ensinar, orientar, supervisionar e proporcionar um ambiente de apoio ao desenvolvimento do autocuidado no tratamento anticoagulante, as quais são eixos estratégicos na Teoria dos Sistemas^(17)^.

Além disso, as ações da intervenção educativa foram estruturadas para aplicação sistemática com base no método dos 5As. O primeiro dia de intervenção (D1) correspondeu à etapa de avaliação do autocuidado, com tempo de duração estimado em cerca de sete minutos; no segundo dia (D2), aplicaram-se as etapas de aconselhamento e acordo, com tempo de aproximadamente de 20 minutos; o terceiro e quarto dias de intervenção (D3 e D4) foram planejados para ofertar assistência às dificuldades no cumprimento do acordo traçado no segundo dia, com tempo aproximado de 15 minutos de duração em cada dia; por fim, no último dia de seguimento (D5), realizou-se o monitoramento do plano de autocuidado com nova avaliação do conhecimento, com tempo de chamada telefônica de oito minutos, aproximadamente. No total, foram realizadas cinco ligações completadas para cada participante, com um tempo estimado de duração de 65 minutos, distribuído entre os contatos telefônicos concluídos.

O Quadro 1 apresenta as ações executadas para promoção do autocuidado dos pacientes em uso de ACOs.

**Quadro 1:** Ações de Autocuidado Apoiado para pacientes anticoagulados de acordo com o método dos 5As e os dias de intervenção, Recife, Pernambuco, Brasil, 2023

DIAS DE INTERVENÇÃO	ETAPA DO MÉTODO 5As	AÇÕES	
**D1**	**Avaliação**	1	Perguntar o que é mais importante para o indivíduo no autocuidado com o tratamento anticoagulante.
		2	Perguntar quais barreiras que o indivíduo percebe para o seu autocuidado no contexto do seu tratamento com ACOs.
		3	Avaliar a importância e o grau de confiança do indivíduo para o desenvolvimento do autocuidado.
**D2**	**Aconselhamento**	1	Informar/reforçar sobre a condição clínica do indivíduo que indica a anticoagulação, sobre o tratamento com a varfarina e sobre o significado dos seus resultados de INR.
		2	Informar ao indivíduo sobre a importância de seguir o tratamento medicamentoso e comportamental da anticoagulação oral.
		3	Informar sobre quais mudanças comportamentais e cuidados são recomendados no contexto da anticoagulação oral.
		4	Encorajar o indivíduo às mudanças comportamentais e a realizar os cuidados necessários no tratamento anticoagulante.
		5	Sugerir formas de operacionalizar as mudanças comportamentais, o autocuidado e o gerenciamento dos ACOs.
	**Acordo**	1	Pactuar com o indivíduo metas específicas para o autocuidado no tratamento anticoagulante.
		2	Estimular o indivíduo a buscar a ajuda de parentes e amigos para o cumprimento das metas.
		3	Elencar e discutir benefícios das metas pactuadas.
		4	Estabelecer, de forma compartilhada, o plano de ações para alcançar as metas definida.
		5	Estabelecer, de forma compartilhada, o grau de confiança da pessoa em alcançar a meta.
**D3 e D4**	**Assistência**	1	Ajudar a pessoa a identificar as barreiras para atingir as metas de autocuidado estabelecidas.
		2	Identificar, com o indivíduo, os recursos existentes na família, na comunidade e em termos de tecnologias disponíveis que possam dar suporte ao autocuidado.
		3	Discutir com o indivíduo sobre o plano de autocuidado, revendo o progresso e reavaliando metas.
		4	Utilizar estratégias motivacionais para continuidade do plano de autocuidado.
**D5**	**Acompanhamento**	1	Monitorar, junto com o indivíduo, o cumprimento do plano de autocuidado.
		2	Avaliar os resultados do nível de conhecimento sobre o tratamento anticoagulante.
		3	Verificar a necessidade de ajustes no plano de cuidados.

*ACOs - anticoagulantes orais; INR - International Normalized Ratio; D - dia.*

*Fonte: Whitlock et al. (2002). Adaptado.*

O intervalo adotado entre cada ligação/dia de intervenção foi de sete a 15 dias, considerando que não se obteve êxito em algumas tentativas de contato. Sendo assim, o tempo médio de acompanhamento dos participantes foi de 38 dias (±8,5), sendo o menor e o maior tempo de seguimento de 28 e 63 dias, respectivamente.

Adicionalmente às ligações telefônicas para os participantes do estudo, os mesmos receberam mensagens de *WhatsApp* ou SMS, sendo consultados sobre a sua predileção por mensagens escritas ou enviadas por áudio. O conteúdo dessas mensagens foi elaborado por dois enfermeiros especialistas e com vivência no ambulatório de anticoagulação. As mensagens ainda foram adaptadas para melhor compreensão dos pacientes, após avaliação e sugestões de uma estudante de graduação do curso de enfermagem.

As mensagens foram personalizadas de acordo as dificuldades no autocuidado identificadas em cada indivíduo no primeiro dia de intervenção (D1). Essa comunicação por mensagem permitiu o reforço das informações recebidas pelos participantes no aconselhamento, com enfoque nas de maior dificuldade de compreensão. As mensagens também reforçaram as metas pactuadas no plano de autocuidado, assim como foram um meio de suporte motivacional para continuidade do autocuidado.

Durante o tempo de seguimento, cada participante recebeu duas mensagens semanais de envios programados, cujo intervalo mínimo foi de três dias. Frequentemente, os indivíduos respondiam ao que lhes era enviado ou disparavam mensagens a fim de esclarecer dúvidas ou de reportar dificuldades. Nessas situações, os mesmos foram respondidos em até 24 horas.

A avaliação do conhecimento foi realizada por meio de instrumento que mede esse desfecho nos pacientes em uso de ACOs, o qual foi devidamente traduzido e validado para o português^(21)^. O questionário é composto por 11 questões, cujas opções de respostas contêm as alternativas “não sabe”, “sabe parcialmente” ou “sabe”, com os valores de zero, meio ponto e um ponto, atribuídos para cada resposta, respectivamente. A partir da quantificação e soma das respostas, obteve-se a seguinte classificação: conhecimento insuficiente (até quatro pontos); conhecimento regular (de cinco a oito pontos); e conhecimento adequado (acima de oito pontos).

#### Análise dos resultados e estatísticas

Para o processamento estatístico, foi utilizado o *Statistical Package for the Social Sciences*, versão 26.0 (IBM Corp., Armonk, NY, EUA), RStudio 2025.09.0 e STATA 12.0. Foram utilizadas ferramentas da estatística descritiva. As diferenças nas características basais entre retidos e excluídos foram avaliadas pelo teste qui-quadrado, para variáveis categóricas, e pelo teste de Mann-Whitney, para variáveis contínuas.

Para comparação das médias pareadas, utilizou-se o método *Bootstrap*, de modo a aumentar a robustez da análise e minimizar possíveis vieses amostrais. Os Intervalos de Confiança de 95% para a diferença das médias foram estimados considerando o procedimento de reamostragem pelo *Bootstrap* BCa (*Bias-Corrected and Accelerated*), o que confere maior precisão estatística, especialmente em amostras de tamanho pequena. A magnitude do efeito da intervenção foi expressa pelo d de Cohen para dados pareados, indicador padronizado da relevância prática das diferenças observadas. O Intervalo de Confiança de 95% para o d de Cohen também foi calculado com base no *Bootstrap* BCa, assegurando maior confiabilidade às estimativas.

Para avaliar a homogeneidade marginal entre as categorias de conhecimento antes e após a intervenção, utilizou-se o teste de Stuart-Maxwell. Esse teste permite verificar se as distribuições marginais de categorias se mantêm equivalentes ao longo do tempo. Todos os testes foram aplicados com nível de significância de 5% (valor de p≤0,05), não sendo realizadas análises adicionais.

## RESULTADOS

Foram acompanhados 25 pacientes em uso crônico de varfarina. Na comparação das características sociodemográficas entre os pacientes retidos (n=25) e excluídos (n=15), não foram observadas diferenças estatisticamente significativas quanto à idade média (58 anos ± 11,7 *versus* 63,60 ± 13,27 anos p=0,154), sexo feminino (60% *versus* 46,7%; p=0,412), raça não branca (80% *versus* 93,3%; p=0,381), estado civil em união conjugal (68% *versus* 66,7%; p=1,000), ausência de ocupação laboral (92% *versus* 73,3%; p=0,174), escolaridade média (6,72 ± 3,47 *versus* 7,73 ± 5,87 anos de estudo; p=0,622) e rendimento *per capita* familiar médio mensal (561,44 ±351,50 *versus* 717,02 ± 612,72 reais; p=0,705).

No que concerne às variáveis clinicas basais, também observou-se homogeneidade das mesmas entre ambos os grupos, como custeio com a medicação (92% *versus* 93,3%; p=1,000), fibrilação atrial como indicação clínica mais frequente para anticoagulação (56% *versus* 60%; p=0,804), tempo mediano de tratamento (4 [p25=2; p75=9] *versus* 3 [p25-2; p75-5] anos; p=0,287), episódios de complicações hemorrágicas (28% *versus* 33,3%; p=0,736) ou tromboembólicas (20% *versus* 26,7%; p=0,705), relato de interrupção do tratamento por conta própria (24% *versus* 20%; p=1,000), recebimento de orientações profissionais sobre a terapia anticoagulante (56% *versus* 53,3%; p=0,870), dúvidas sobre o tratamento (36% *versus* 53,3%; p=0,283) e escore médio de conhecimento sobre o tratamento (5,24 ± 1,38 *versus* 4,63 ± 2,01 %; p=0,249).

O conhecimento dos participantes foi abordado nos dois momentos de aferição desse desfecho (antes e após a intervenção). A Tabela 1 demonstra o conhecimento total, parcial e a ausência de conhecimento sobre pontos relevantes ao tratamento anticoagulante. O conhecimento deficitário mais frequente antes da intervenção foi sobre os efeitos colaterais da medicação, os cuidados necessários ao tratamento, valor do INR alvo, fatores que interferem a estabilidade do INR, consequência da não utilização do medicamento, assim como a indicação clínica para o tratamento. A realização do acompanhamento conduziu a menores percentuais no déficit do conhecimento.

**Tabela 1:** Conhecimento dos pacientes sobre a terapia com anticoagulantes orais antes e após a intervenção (N = 25), Recife, Pernambuco, Brasil, 2023

QUESTÕES	Sabe n (%)		Sabe parcialmente n (%)		Não sabe n (%)	
	Antes	Depois	Antes	Depois	Antes	Depois
1. Qual o nome do anticoagulante que você está tomando?	21 (84)	23 (92)	01 (04)	-	03 (12)	02 (08)
2. Você sabe para que serve esse medicamento?	15 (60)	21 (84)	08 (32)	03 (12)	02 (08)	01 (04)
3. Você sabe por que está tomando esse medicamento?	09 (36)	15 (60)	06 (24)	05 (20)	10 (40)	05 (20)
4. Você sabe dizer quais são os efeitos colaterais do anticoagulante? (ao menos 1)	01 (04)	16 (64)	01 (04)	-	23 (92)	09 (36)
5. Qual a dose do ACO que você está tomando agora? Dizer.	20 (80)	23 (92)	02 (08)	01 (04)	03 (12)	01 (04)
6. Há quanto tempo você está tomando ACO?	16 (64)	16 (64)	08 (32)	07 (28)	01 (04)	02 (08)
7. O que pode acontecer se você não tomar o ACO?	07 (28)	15 (60)	08 (32)	01 (04)	10 (40)	09 (36)
8. Qual o seu INR alvo?	10 (40)	15 (60)	01 (04)	07 (28)	14 (56)	03 (12)
9. Você sabe que fatores podem interferir nos níveis de INR? (ao menos 1)	11 (44)	18 (72)	01 (04)	01 (04)	13 (52)	06 (24)
10. Você sabe que cuidados tem que ter por estar usando o ACO? (ao menos 2)	01 (04)	12 (48)	10 (40)	11 (44)	14 (56)	02 (08)

*ACO - anticoagulante oral; INR - International Normalized Ratio.*

Conforme demonstrado na Tabela 2, a média dos escores do conhecimento dos participantes sobre o tratamento aumentou em mais de dois pontos após a intervenção de enfermagem. Essa diferença apresentou significância estatística (p<0,001) com tamanho de efeito expressivo (d = 1,53; IC95%: 1,07 - 2,04). Ao comparar também as categorias do desfecho antes e depois da intervenção, percebe-se migração positiva entre as categorias do conhecimento (de insuficiente para regular e satisfatório). A análise pelo teste de homogeneidade marginal de Stuart-Maxwell evidenciou diferença estatisticamente significativa entre as distribuições marginais dos dois momentos (p<0,001), indicando que o perfil de conhecimento dos participantes sofreu modificação relevante ao longo do tempo.

**Tabela 2:** Escore do conhecimento sobre a terapia anticoagulante antes e após a intervenção (N = 25), Recife, Pernambuco, Brasil, 2023

CONHECIMENTO								
	**Antes**		**Depois**					
	**n (%)**	**Escore médio** **(±DP)**	**n (%)**	**Escore médio** **(±DP)**	IC95% da Diferença ^**^	Valor de *p* ^*^	**d de Cohen (pareado)**	**IC95%** d Cohen ^**^
Insuficiente	09 (36)	5,24 (±1,38)	-	7,74 (±1,24)	1,90 - 3,14	**<0,001**	1,53	1,07 - 2,04
Regular	16 (64)		14 (56)					
Satisfatório	-		11 (44)					

* Comparação de médias pareado pelo método Bootstrap;

** IC95% (Bootstrap BCa); IC95% - Intervalo de Confiança de 95%; DP - desvio padrão.

Algumas das ações de autocuidado sugeridas aos pacientes são: organização da rotina diária, assim como uso de alarmes e ajuda familiar para evitar esquecimentos de ingestão do medicamento; aumento do conhecimento sobre a interação alimentar, medicamentosa e fitoterápica; realização do monitoramento regular do INR com presença na consulta ou teleconsulta; planejamento alimentar; utilização de equipamentos de proteção em atividades de risco de corte ou sangramento; e diálogo com a equipe de saúde para buscar alternativas possíveis para o tratamento de saúde diante das dificuldades financeiras em adquirir os medicamentos.

## DISCUSSÃO

Nos resultados encontrados sobre o conhecimento dos aspectos relacionados ao tratamento com ACOs, foi possível observar que, após a intervenção, alcançou-se conhecimento adequado na maioria das questões, com maior destaque para o aumento do conhecimento sobre os efeitos colaterais da medicação e os cuidados necessários por usar ACOs.

Ao comparar o escore médio do desfecho antes e após a intervenção, verifica-se impacto positivo no conhecimento terapêutico. Estudo quase-experimental, realizado em ambulatório de anticoagulação, verificou que, após acompanhamento com visitas periódicas e realização de aconselhamento sobre a medicação anticoagulante, houve um aumento significativo do escore de conhecimento sobre o tratamento^(19)^.

Intervenções educacionais remotas com o uso de tecnologias também têm sido possibilidades viáveis e com impacto relevante no conhecimento dos pacientes sobre sua terapia. Estudo de revisão sistemática evidenciou melhora nos níveis de conhecimento dos pacientes que receberam instruções educacionais e de autogestão no contexto da anticoagulação por meio de aplicativos móveis em *smartphones* ou *tablets* ^(11)^.

Adicionalmente, cabe ressaltar o destaque que a telessaúde ganhou, especialmente após o contexto pandêmico da COVID-19, demonstrando ser possível em muitas situações garantir a continuidade da assistência à saúde aos pacientes de forma remota. Esse recurso é capaz de gerar impactos positivos nos resultados clínicos, além do acesso mais flexível ao sistema de saúde. Nesse espectro, há projeções para permanência da telessaúde e sua provável expansão como formato complementar aos atendimentos presenciais dos pacientes^(22)^.

Sendo assim, tem se tornado cada vez mais comum o relato do uso das tecnologias aplicadas ao tratamento anticoagulante, com resultados satisfatórios tanto na continuidade dos cuidados de saúde quanto na melhora dos níveis de conhecimento, adesão terapêutica, satisfação e nos resultados clínicos. Essas abordagens tecnológicas se dão por meio de programas de educação em saúde por teleconsulta, mensagens de texto e aplicativos móveis^(12,13)^.

As estratégias de repetição do aconselhamento no âmbito da educação em saúde, inclusive através de mensagens de texto, têm sido citadas na literatura como formas de maior consolidação do conhecimento dos pacientes^(6,12)^. Dessa maneira, pode-se inferir que, possivelmente, tanto o reforço telefônico das orientações sobre o tratamento com a varfarina quanto o envio periódico semanal de mensagens de texto aos pacientes neste estudo foram fatores contribuintes para o alcance de níveis de conhecimento mais satisfatórios.

Nessa perspectiva, uma experiência educacional e participação ativa do paciente, com discussão de experiências negativas acerca da medicação e do tratamento, podem fortalecer o conhecimento e adesão terapêutica no tratamento anticoagulante^(8)^.

No que diz respeito à abordagem da literatura ao AA na anticoagulação, em estudos internacionais, é perceptível que a forma com que é apresentada essa temática geralmente traz o autocuidado para a finalidade de autotestagem e automanejo das doses do medicamento^(14,23)^. Isso difere da abordagem brasileira, cujo enfoque de promoção do autocuidado está mais direcionado aos cuidados gerais sem envolver aspectos mais complexos do tratamento^(24)^.

Quanto à utilização do AA de forma sistemática para pacientes anticoagulados, foram encontradas duas pesquisas com essa abordagem. Estudo desenvolvido na Turquia utilizou o AA sistematizado no método dos 5As dentro de um programa de atenção domiciliar desenvolvido por enfermeiros aos pacientes em uso de varfarina, obtendo efeitos positivos no autogerenciamento e na satisfação dos mesmos^(14)^.

Outro estudo foi desenvolvido no Brasil, e correspondeu à construção e validação de protocolo para promoção da mudança de comportamento em pacientes em uso de varfarina, buscando participação ativa dos mesmos no autocuidado. Embora não tenha referido como base o método dos 5As, o protocolo criado inclui algumas das etapas do referido método, como avaliação do paciente com definição dos problemas, pactuação de metas, elaboração de plano de cuidados e assistência ao cumprimento das metas pactuadas no plano^(24)^.

Sob a ótica da TGA, Orem menciona sobre os requisitos de autocuidado específicos diante dos desvios de saúde. Essa especificidade foi pensada para pessoas que possuem alguma doença, deficiência ou distúrbio com necessidades peculiares de cuidado^(17)^. A partir desse ponto de vista, compreende-se que as pessoas com condições crônicas de saúde comumente necessitam de uma rotina específica de autocuidado, considerando as demandas que surgem no curso de sua doença^(25)^. Os pacientes anticoagulados também requerem a dispensação de cuidados específicos, tendo em vista a complexidade do seu tratamento.

Ademais, cabe ressaltar a importância do vínculo entre a equipe de saúde, o indivíduo e seus familiares como fio condutor dentro do processo de promoção do autocuidado e de apoio educativo do enfermeiro para o alcance de resultados favoráveis. Para tanto, construir um vínculo implica responsabilidade entre os profissionais e o indivíduo, resultante da coexistência de elementos como comunicação eficaz, estabelecimento de confiança e relações afetivas^(25)^.

### Limitações do estudo

O presente estudo apresentou algumas limitações no que diz respeito ao tamanho amostral, pois os pesquisadores se depararam com a saturação dos indivíduos elegíveis dentro da população estudada, além da inviabilidade em randomizar a amostra.

Ademais, é pertinente destacar os vieses que podem afetar a validade interna do estudo. Alguns desses vieses são: regressão à média, minimizada pela presença de escores iniciais de conhecimento variados entre os pacientes; efeito de teste, atenuado pelo intervalo de tempo mínimo de quatro semanas entre as avaliações e pela característica conceitual do questionário; fidelidade na implementação da intervenção, a qual foi assegurada pelo seguimento de um protocolo de condução da intervenção educativa e por monitoramento externo; e viés de história e maturação, que pode ter influenciado os resultados positivos da intervenção, apesar de o curto tempo de seguimento sugerir baixo impacto na maturação natural dos participantes.

No que diz respeito à validade externa, a generalização dos resultados deve considerar a frequência e padronização da intervenção conforme protocolo, a linguagem da intervenção educativa adaptada à escolaridade e cultura local, o acesso aos recursos tecnológicos para comunicação, e a condução do estudo por equipe vinculada a hospital de referência em cardiologia, cujo suporte é diferente do ofertado nos serviços de menor complexidade.

Diante do exposto, recomenda-se a realização de novos estudos com delineamentos randomizados e em mais de um serviço de saúde que testem a aplicação do AA em pacientes anticoagulados e seus benefícios clínicos.

### Contribuições para as áreas da saúde e da enfermagem

Os achados da presente pesquisa, no que dizem respeito ao impacto positivo do AA por teleconsulta conduzida por uma profissional enfermeira, poderão servir de subsídio para aplicação desse modelo de atenção e seu aprimoramento no acompanhamento ambulatorial dos indivíduos em uso de ACOs, bem como no contexto de outras condições crônicas.

Além disso, ampliam as possibilidades de atuação da enfermagem, através das tecnologias de comunicação, para prestar uma assistência qualificada e com otimização dos recursos disponíveis. A manutenção e fortalecimento do vínculo com o paciente por telessaúde evidenciaram, portanto, o potencial dessa ferramenta na assistência ao indivíduo em tratamento com anticoagulantes.

## CONCLUSÕES

A intervenção educativa de enfermagem teve impacto positivo no conhecimento dos pacientes sobre a terapia anticoagulante, indicando sua efetividade. Dessa forma, o estudo aponta para a potencialidade de êxito na condução de programas educativos em saúde, liderados por enfermeiros e realizados remotamente junto aos pacientes anticoagulados.

## Data Availability

Os dados de pesquisa estão disponíveis no corpo do artigo.
